# Supplementary material for: The HAC1 histone acetyltransferase promotes leaf senescence and regulates the expression of ERF022
Source: Plant Direct. 2019 Aug 27;3(8):e00159. doi: 10.1002/pld3.159 (PMC6710649; doi:10.1002/pld3.159)
Supplement: Supplementary file 1 [file PLD3-3-e00159-s002.pdf]

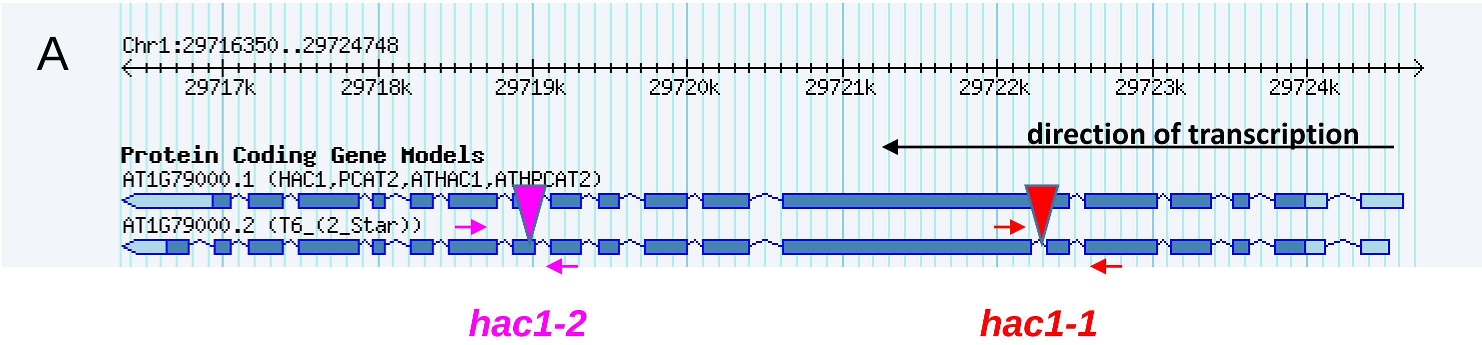

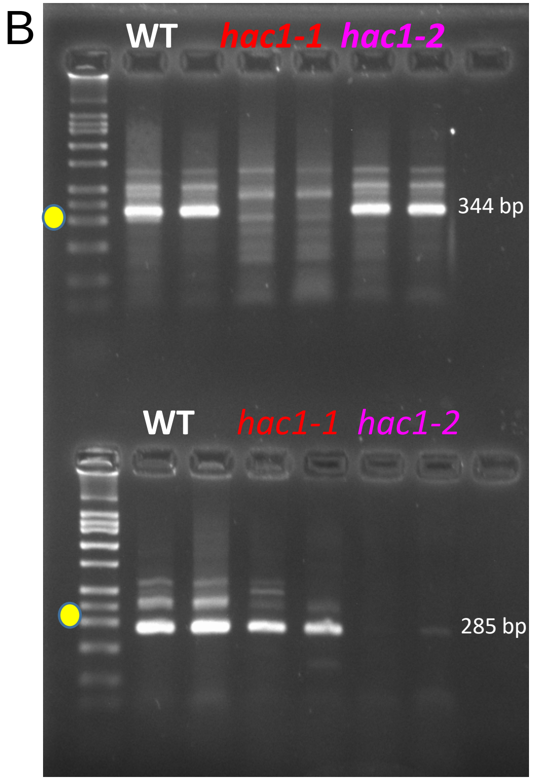

Supplemental Figure 1: No full-length transcripts are produced in *hac1* alleles. A) Gene models for both *HAC1* isoforms, AT1G79000.1 and AT1G79000.2, are displayed with the locations of the T-DNA insertions for *hac1-1* (SALK_080380, intron 6) and *hac1-2* (SALK_136314, exon 12). The direction of transcription and the F/R primers used to amplify cDNA are shown. B) The top panel shows amplification of two cDNAsamples from two different plants for each genotype amplified with the *hac1-1* F/R primers. The bottom panel shows the same cDNA samples amplified with the *hac1-2* F/R primers. The allele-specific primers do not amplify their respective alleles, however partial mRNAs are produced downstream of the *hac1-1* T-DNA and upstream of the *hac1-2* T-DNA insertion.
