# Supplementary material for: The HAC1 histone acetyltransferase promotes leaf senescence and regulates the expression of ERF022
Source: Plant Direct. 2019 Aug 27;3(8):e00159. doi: 10.1002/pld3.159 (PMC6710649; doi:10.1002/pld3.159)
Supplement: Supplementary file 3 [file PLD3-3-e00159-s003.pdf]

Reviewer #1:

Hinckley et al. investigated the effect of HAC1 on leaf senescence based on combination of genetic evidence and high-throughput technologies. The genetic evidence is relatively solid, but the presentation of the details about high-throughput data is limited, which restricted the evaluation and further application.

Specifically, the major comments include
1. High-through data need to be deposited to a public database and the accession number needs to be provided.

Line 463-464: RNA-seq data files are available at NCBI GEO under accession GSE134176. ChIP-seq data files are available under accession PRNJA552590 in the short read archive (SRA).

2. Whether the replicates of high-throughput data are biological replicates or technical replicates?

RNA-seq replicates were from plants that were grown together. Leaves were harvested and divided into three pools that were processed separately. I think this is considered a technical replicate.

Line 121 "Indicated leaves were harvested, divided into three pools for three technical replicates, and stored in liquid nitrogen."

3. The detailed statistics, expression value, differential expression result, ChIP-seq density and quantitative difference of histone modification between WT and mutant need to be listed in supplementary tables, at least for genes with differential expression and different level of histone modification.

An excel file with 6 sheets will be included as Supplemental Data. The title of the tables are listed on lines 434-443.

Supplemental Table 1: ChIP-seq identified 976 loci with reduced H3K9ac marks in both *hac1* alleles. Read counts and statistics are shown for gene loci with reduced H3K9ac marks.

Supplemental Table 2: ChIP-seq identified 368 loci with reduced H3K4me3 marks in both *hac1* alleles. Read counts and statistics are shown for gene loci with reduced H3K4me3 marks.

Supplemental Table 3: RNA-seq identified 143 loci with reduced mRNA levels in both *hac1* alleles. Reach counts and statistics are shown for gene loci with reduced gene expression.

Supplemental Table 4: GO enrichment analysis results and statistics.

Supplemental Table 5: The 43 genes with lower gene expression and reduced H3K9ac marks in both *hac1* alleles are listed.

Supplemental Table 6: PCR primers used in this study.

Importantly, when making these tables, it was discovered that some of the H3K4me3 peaks did not surpass the 1.4-fold cutoff between WT and both *hac1* alleles, and these were removed from the

analysis. This dropped the number of decreased H3K4me3 peaks from 548 to 366. A revised Venn Diagram is now shown in Figure 3. I apologize for this oversight in the original submitted version of the manuscript.

4. The bioinformatics part in Methods described the packages used for GO enrichment and pathway analysis, but no specific result or figure was presented in the manuscript.

Line 178-185 discuss the GO terms and a new Supplemental Table 5 lists Pathway and GO Biological Process results with q-value or FDR. The Panther GO Enrichment tool, available through www.arabidopsis.org was used for GO enrichment as it has been recently updated (2/2019). This change is indicated in the Methods (line 134-135).

Minor:
1. page number need to be inserted Done
2. "MANorm " (line 130) need to be changed to "MAnorm" Done

Reviewer #2:

In this manuscript, the involvement of the HAC1 histone acetyltransferase in leaf senescence was investigated. It was shown that two Arabidopsis hac1 alleles display delayed age-related developmental senescence. Using a combination of ChIP-seq for H3K9ac and RNA-seq for gene expression, 44 potential HAC1 targets during age-related developmental senescence were identified. Genetic analysis demonstrated that one of these potential targets, ERF022, is a positive regulator of leaf senescence. ERF022 is regulated additively by HAC1 and MED25. It was proposed that MED25 may recruit HAC1 to the ERF022 promoter to increase its expression in older leaves. This is an interesting paper revealing a possible mechanism of epigenetic regulation in leaf senescence through regulation of ERF022 by HAC1. However, I think the following comments need to be addressed to improve the manuscript.

1. Please add a figure to show the structure of the HAC1gene, the T-DNA insertion sites of hac1 and hac2 mutants, and the positions of the primers used for PCR and RT-PCR analysis. In addition, please also show the data indicating that hac1 and hac2 mutants are indeed gene knock-out mutants.

This figure has been added to Supplemental Figure 1, panel A with a revised Figure Legend:
Supplemental Figure 1: No full-length transcripts are produced in *hac1* alleles. A) Gene models for both *HAC1* isoforms, AT1G79000.1 and AT1G79000.2, are displayed with the locations of the T-DNA insertions for *hac1-1* (SALK_080380, intron 6) and *hac1-2* (SALK_136314, exon 12). The direction of transcription and the F/R primers used to amplify cDNA are shown. B) The top panel shows amplification of two cDNA samples from two different plants for each genotype amplified with the *hac1-1* F/R primers. The bottom panel shows the same cDNA samples amplified with the *hac1-2* F/R primers. The allele-specific primers do not amplify their respective alleles, however partial mRNAs are produced downstream of the *hac1-1* T-DNA and upstream of the *hac1-2* T-DNA insertion.

2. Figure 1

Please indicate how many plants for each line were used for data analysis. For Figure 1B, the authors need to analyze gene expression in both hac1 and hac2 mutants.

The legend for Figure 1 was modified.

For 1A: n = 6 individual plants for each genotype and time point (line 391).

Figure 1B was changed to show *NIT2* expression at 45d from a different biological replicate. The Figure legend now reads, "B) RNA was extracted from WT and both *hac1* alleles at 45 days from leaf 6 of a distinct biological replicate than that used in panel A, and *NIT2* gene expression was measured by real-time qPCR (n = 3)." (Lines 392-394)

For 1C: "Leaf 5 was removed from plants grown for 21 days (n = 24 per genotype), and floated on water in the dark for the indicated number of days to observe dark-induced senescence. Six leaf discs were removed from dark treatment at each time point, and chlorophyll was measured. (Lines 394-397).

3. Page 6, line 165 - "H3K4me3 modifications were similarly affected, with 548 loci showing a loss and only 33 loci showing a gain of H3K4me3 marks".

Please explain why H3K4me3 modifications are affected in hac1-1 and hac1-2 mutants.

The following was added to lines 171-172, "In addition, histone acetylation and the H3K4me3 mark are shared components of one of four chromatin signatures in Arabidopsis (Roudier et al., 2011)."

4. Figure 4A-B and Figure 5A-B

Please indicate how many plants for each line were used for data analysis.

We now indicate that n=6 single hole punches from 6 individual plants for chlorophyll and protein (lines 103 and 109) and for gene expression, "Each leaf sample was from one individual plant." (Line 117-118)

5. Page 7, line 234 - "These data suggest that MED25 guides HAC1 to histones at the ERF022 locus to direct histone acetylation for increased chromatin accessibility".

This sentence was changed to " These data suggest that MED25 may guide HAC1 to histones at the *ERF022* locus to direct histone acetylation for increased chromatin accessibility." (line 241).

There is no direct evidence to support that either MED25 or HAC1 targets directly at the ERF022 locus. To analyze whether MED25 and HAC1 target directly at the ERF022 locus, the authors need to carry our ChIP experiment using the MED25 or HAC1 antibody.

Yes, we definitely agree. These are the experiments we are planning to do. We have been trying to obtain HA-tagged or GFP-tagged HAC1 or MED25 lines from other groups who have published work, but seeds have not been sent nor are they available at the ABRC. A recent paper (doi: 10.1104/pp.19.00511) listed the peptide used to generate an anti-HAC1 antibody, and we plan on generating HA-tagged MED25 and Flag-tagged ERF022 to see if we can isolate a ternary complex. This is beyond the scope of

this manuscript, but the suggestion of next steps is hugely appreciated.

6. Although the expression of ERF022 is affected in hac1 and hac2 mutants, more evidences are required to support the claim that the HAC1 histone acetyltransferase promotes leaf senescence via regulation of ERF022. I suggest the authors change the title of the manuscript to "The HAC1 histone acetyltransferase promotes leaf senescence and regulates the expression of ERF022".

The title has been changed, as suggested.
